# Supplementary material for: Effective implementation of L0-regularised compressed sensing with chaotic-amplitude-controlled coherent Ising machines
Source: Sci Rep. 2023 Sep 26;13:16140. doi: 10.1038/s41598-023-43364-8 (PMC10522662; doi:10.1038/s41598-023-43364-8)
Supplement: Supplementary file 1 — Supplementary Information. [file 41598_2023_43364_MOESM1_ESM.pdf]

Effective implementation of l_0-Regularised Compressed Sensing with Chaotic-Amplitude-Controlled Coherent Ising Machines

Supplementary Information

Mastiyage Don Sudeera Hasaranga Gunathilaka, Satoshi Kako, Yoshitaka Inui, Kazushi Mimura, Masato Okada, Yoshihisa Yamamoto, and Toru Aonishi

Supplementary Note 1: Performance dependence on system size

This section compares how the performance of each model differs as the system size increases. In this case, N is in the range of [200, 5000], with compression $\alpha = 0.6$ and sparseness $a = 0.2$. Fig. S1 illustrates the acquired results. Black, blue and red marks indicate the log average RMSE values acquired for 10 random hamiltonian simulations for OL-CIM-CDP, CAC-CIM-CDP (Wigner) and CAC-CIM-CDP (Positive-P) respectively.

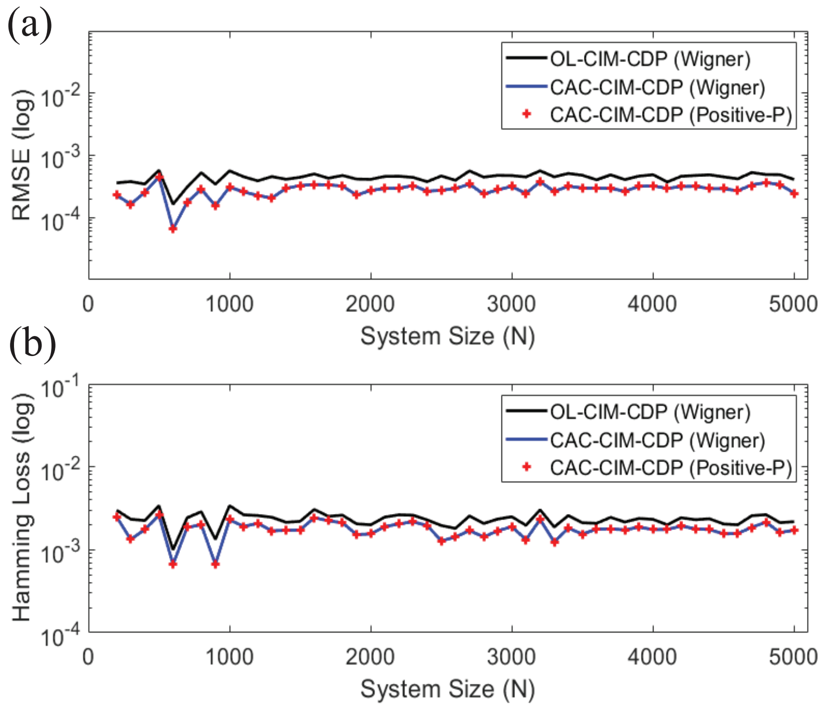

Figure S1: **Performance on different System Sizes**

(a) Log average RMSE values for 10 random Hamiltonians for each system size. For CAC-CIM-CDP (Wigner and Positive-P) $j = 1.0$, and $\beta = 1.0$ were used. η_{init} and η_{end} assigned as 0.9 and 0.01 respectively. For OL-CIM-CDP η_{init} and η_{end} were set as 0.6 and 0.01 respectively. **(b)** Log average Hamming loss values for 10 random Hamiltonians for each system size calculated according to Eq. (S1) which calculates the average support estimation accuracy.

As a measure of model performance, we have used average RMSE (Root Mean Squared Error) and average Hamming loss of support estimation from CIM. Using the following formula (S1), we calculated the hamming loss.

$$Hamming\ Loss = \frac{1}{N}\left(\sum_{r=0}^{N}|\sigma_r^{CIM} - \xi_r|\right). \tag{S1}$$

Here ξ_r corresponds to the correct support while σ_r^{CIM} is the estimation produced by CIM models. Based on the RMSE values in the top graph of Fig. S1, we can state that even when the size of the system changes, CAC-CIM-CDP models maintain a lower average RMSE value than OL-CIM-CDP. In addition, the corresponding hamming loss values (bottom graph of Fig. S1) are consistent also with the RMSE results. This suggests that CAC-CIM-CDP has performed a more accurate sparse reconstruction.

Thus, we can conclude that CAC-CIM-CDP provides better results than OL-CIM-CDP, regardless of system size.

Supplementary Note 2: Performance difference on relatively harder problem instances

Here we discuss the observation we had on the amplitude evolution of CAC-CIM-CDP models when the a/α ratio is higher (after exceeding the critical threshold).

The difference in performance is clear in normalised measured-amplitude $g\tilde{\mu}_r$ and error e_r as well. In Fig. S2 and Fig. S3 $N = 2000$ system is considered with $\alpha = 0.6$. Fig. S2a and Fig. S2b $a = 0.2$ is used while Fig. S2c and Fig. S2d use $a = 0.4$. It is clear that in the $a = 0.4$ case, the amplitude evolution is more chaotic compared to $a = 0.2$ in both models. The corresponding e_r evolution is illustrated in Fig. S3. Even though $a = 0.2$ has identical evolution for CAC-CIM-CDP, with $a = 0.4$ the evolution is slightly different.

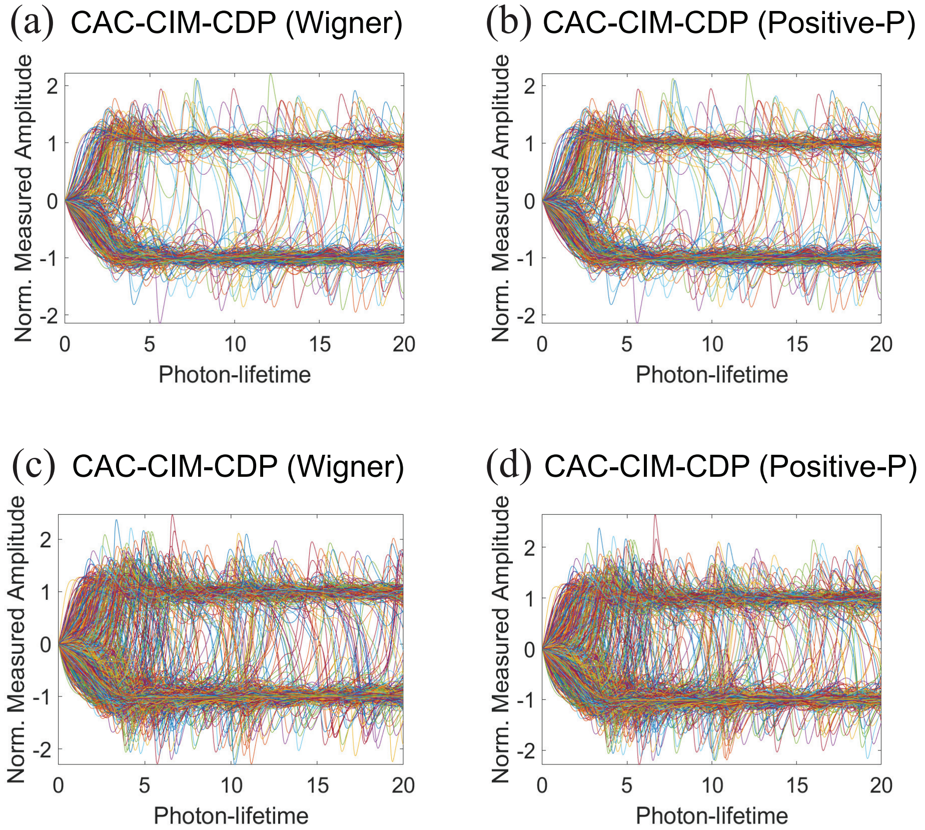

Figure S2: **Normalised measured amplitude $g\tilde{\mu}_r$ evolution for easy and harder problem instances**

(a) and **(b)** indicates the normalised amplitude $g\tilde{\mu}_r$ evolution of CAC-CIM-CDP (Wigner and Positive-P) with $a = 0.2$ and $\alpha = 0.6$. **(c)** and **(d)** indicates the normalised amplitude $g\tilde{\mu}_r$ evolution of CAC-CIM-CDP (Wigner and Positive-P) with $a = 0.4$ and $\alpha = 0.6$.

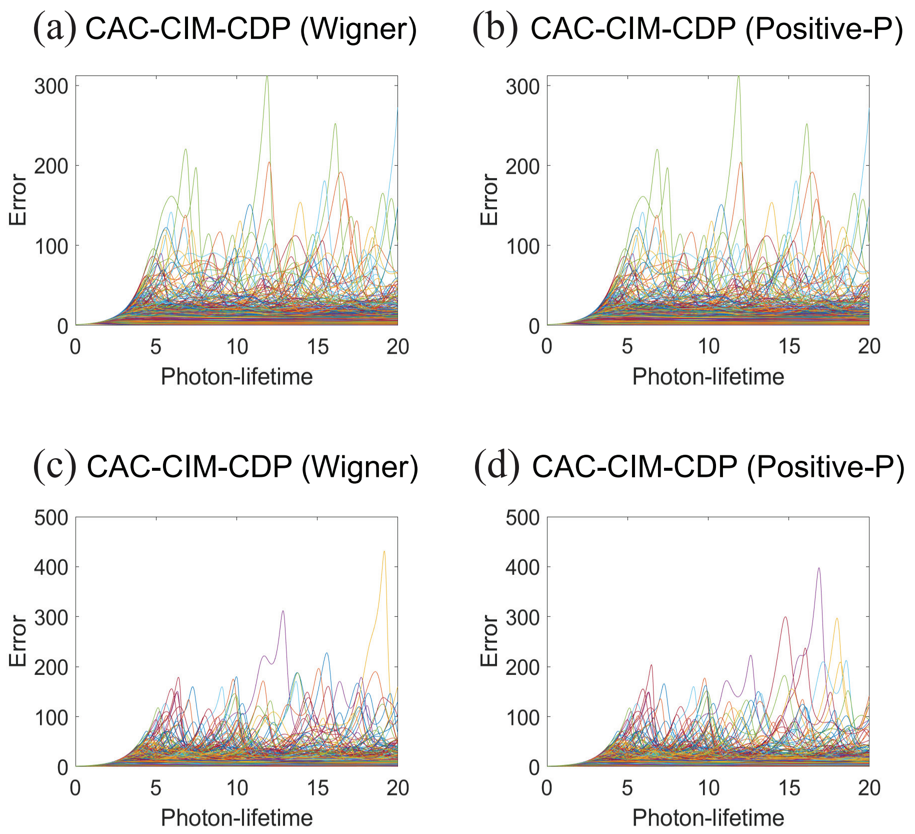

Figure S3: **Error e_r evolution for easy and harder problem instances**

(a) and **(b)** indicates the error amplitude e_r evolution of CAC-CIM-CDP (Wigner and Positive-P) with $a = 0.2$ and $\alpha = 0.6$. **(c)** and **(d)** indicates the error amplitude e_r evolution of CAC-CIM-CDP (Wigner and Positive-P) with $a = 0.4$ and $\alpha = 0.6$.

Supplementary Note 3: Performance with larger saturation parameters

With the same parameters used for CAC-CIM-CDP (Wigner and Positive-P), here we conducted an experiment on how the performance varies when the saturation parameter g^2 changes. Parameter g^2 corresponds directly to the quantum noise present in the CIM system. The higher the g^2, the more quantum noise is present. In the MRI experiments mentioned previously, $g^2 = 10^{-7}$ was used. Which is quite small. In Fig. S4, we compare CIMs performance with $g^2 = 10^{-2}$, $g^2 = 10^{-3}$, and $g^2 = 10^{-7}$. Red lines indicate the CAC-CIM-CDP (Wigner) and the blue dashed line corresponds to the CAC-CIM-CDP (Positive-P) model. Vertical lines are the error bars for each threshold η value.

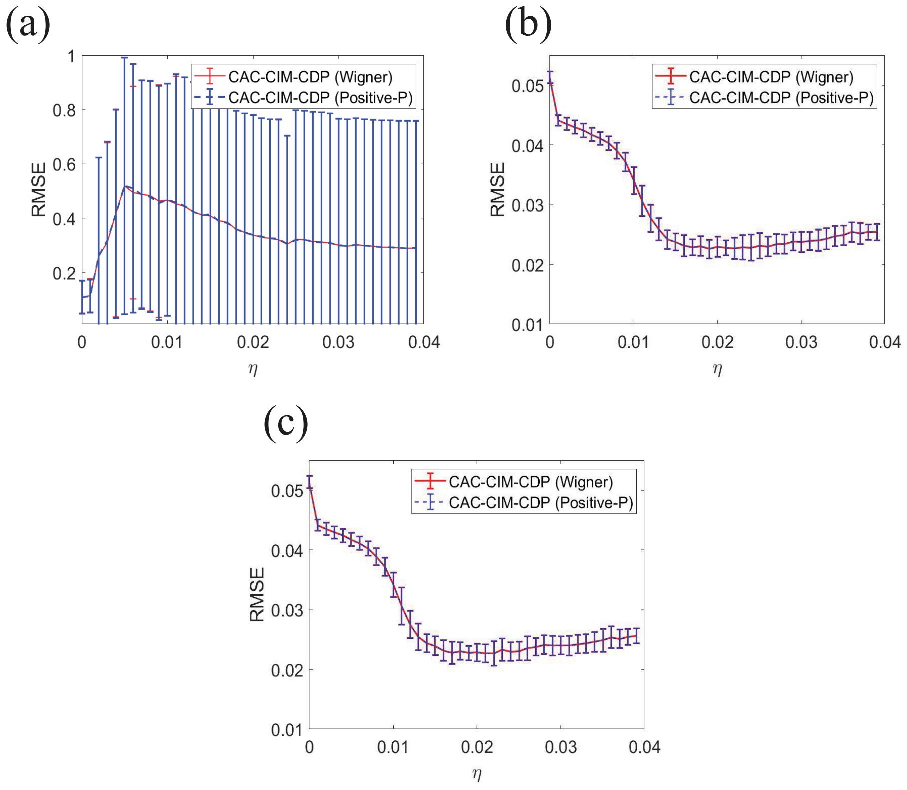

Figure S4: **Performance of CAC-CIM-CDP models with large quantum noise present**

(a), **(b)**, and **(c)** illustrates the performance of CAC-CIM-CDP models when g^2 is 10^{-2}, 10^{-3} and 10^{-7} respectively for 64×64 image reconstruction.

Results indicate that both CAC-CIM-CDP models tend to perform similarly in all the cases. Yet when $g^2 = 10^{-2}$, reconstruction fails. Compared to other g^2 values (which have better reconstruction), it implies that for CIM performance there is a threshold saturation parameter value.

Supplementary Note 4: Performance change in sparseness

Here we consider different sparseness a values. For the simulations 64×64 images were used.

Reconstruction becomes harder when the a/α ratio becomes larger. In Fig. S5, we showcase the results on how the performance of LASSO, OL-CIM-CDP and the two CAC-CIM-CDP models change when a is increased for a constant α value with respect to η. The green boxplot indicates the CAC-CIM-CDP (Wigner) and the blue boxplot corresponds to the CAC-CIM-CDP (Positive-P) model. The black line and red boxplot indicate the LASSO algorithm and OL-CIM-CDP respectively. Based on different threshold values, box plots illustrate the maximum, minimum, 25th percentile (bottom edge), 75th percentile (top edge), and median (central horizontal line) of RMSEs for each model. In the markers, outliers are identified.

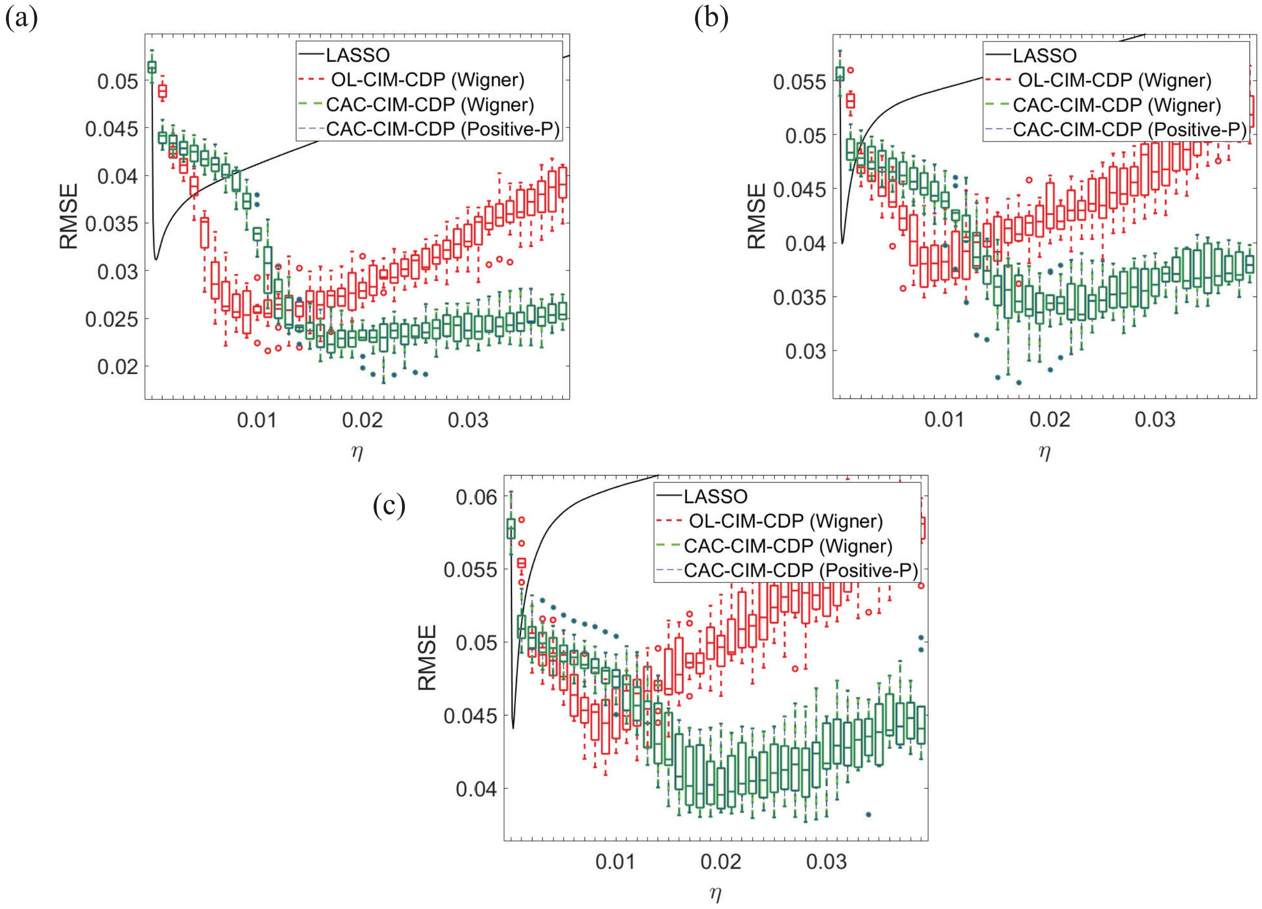

Figure S5: **Average performance of LASSO, OL-CIM-CDP, CAC-CIM-CDP (Wigner) and CAC-CIM-CDP (Positive-P) models when sparseness varies for 64×64 MRI image**

(a), **(b)**, and **(c)** correspond to the reconstructions obtained from LASSO, OL-CIM-CDP, and CAC-CIM-CDP (Positive-P) with sparseness values 0.212, 0.255, and 0.280 respectively. For every model compression ratio was 0.4. The black line indicates the performance on LASSO while the red boxes correspond to OL-CIM-CDP. Green and blue boxes indicate the performance on CAC-CIM-CDP Wigner and Positive-P respectively. For different threshold values, the graphs illustrate the maximum, minimum, 25-th percentile (bottom edge), 75-th percentile (top edge), and median (central horizontal line) of RMSEs for each model with box plots. The markers indicate the outliers.

With the above results, it is clear that CAC-CIM-CDP models tend to perform better than OL-CIM-CDP. However, when a increases the reconstruction accuracy worsens in all three CIM-CDP models and LASSO. Even though the accuracy deteriorates, CAC-CIM-CDP models still perform better than OL-CIM-CDP. This indicates that even though the problem becomes more challenging, CAC-CIM-CDP is still a better CIM model than the OL-CIM-CDP for l_0-regularised CS.
